# Machine Learning Techniques to Identify Antimicrobial Resistance in the Intensive Care Unit

**DOI:** 10.3390/e21060603

**Published:** 2019-06-18

**Authors:** Sergio Martínez-Agüero, Inmaculada Mora-Jiménez, Jon Lérida-García, Joaquín Álvarez-Rodríguez, Cristina Soguero-Ruiz

**Affiliations:** 1 Department of Signal Theory and Communications, Telematics and Computing Systems, Rey Juan Carlos University, Madrid 28943, Spain; 2 Intensive Care Department, University Hospital of Fuenlabrada, Madrid 28942, Spain

**Keywords:** machine learning, clinical data, antibiogram, feature selection, correspondence analysis, culture, antimicrobial resistance, bacteria, intensive care unit

## Abstract

The presence of bacteria with resistance to specific antibiotics is one of the greatest threats to the global health system. According to the World Health Organization, antimicrobial resistance has already reached alarming levels in many parts of the world, involving a social and economic burden for the patient, for the system, and for society in general. Because of the critical health status of patients in the intensive care unit (ICU), time is critical to identify bacteria and their resistance to antibiotics. Since common antibiotics resistance tests require between 24 and 48 h after the culture is collected, we propose to apply machine learning (ML) techniques to determine whether a bacterium will be resistant to different families of antimicrobials. For this purpose, clinical and demographic features from the patient, as well as data from cultures and antibiograms are considered. From a population point of view, we also show graphically the relationship between different bacteria and families of antimicrobials by performing correspondence analysis. Results of the ML techniques evidence non-linear relationships helping to identify antimicrobial resistance at the ICU, with performance dependent on the family of antimicrobials. A change in the trend of antimicrobial resistance is also evidenced.

## 1. Introduction

Antimicrobials have revolutionized the healthcare system since penicillin was discovered in 1928, transforming medicine and saving millions of lives. Currently, the efficacy of antimicrobials in treatments of bacterial infections is decreasing, mainly due to their excessive and inadequate use [1].

As a result, bacteria are developing resistance to antimicrobial agents. Antimicrobial resistance can be defined as the capacity of bacteria to withstand the effects of a harmful chemical agent designed to damage it [2]. That means that the bacteria are not killed and continue alive.

Bacteria may resist antibiotic action by using several mechanisms. Some bacterial species are innately resistant to one class of antibiotics . In such cases, all clinical samples of those bacterial species are likewise resistant to all the members of those antibacterial classes. An increasing concern is bacteria that become resistant: e.g., initially susceptible bacteria become resistant to antibiotics and consequently disseminate under the selective pressure of these antibiotics. Several mechanisms of antimicrobial resistance are readily spread to a variety of bacterial genera. Resistance is not necessarily limited to a single family of antibiotics. It can apply, simultaneously, to many chemically unrelated compounds to which the bacteria have never been exposed, this is termed multi-drug resistance [3].

Antibiotic resistance has increased worldwide during the last decades, leading to treatment failures in infectious diseases. Infections caused by antibiotic-resistant bacteria are difficult, and sometimes impossible to treat. In most cases, antibiotic-resistant infections require longer hospital stays, additional follow-up doctor visits, and costly and toxic alternatives. Infections are a major cause of admission in hospitals, and are the most frequent complication in hospitalized patients.The intensive care units (ICU) are the units of the hospital where the most seriously ill patients are. Due to their pathology and fragility, patients in ICU require advanced means of monitoring and life support. Infections in these patients are of special severity and an important cause of mortality. Septic shock and severe sepsis are the most serious clinical form of infection in the ICU setting, and can reach a mortality rate up to 80% [4,5,6,7,8]. Currently, many of the efforts of hospitals are focused on an early detection of serious infections. It has been shown that the sooner the correct antibiotic treatment is started, the lower the mortality [9,10]. If there is a suspicion of a serious infection, antibiotic treatment is established empirically, taking into account the possible infectious focus, the most probable germ or germs and the susceptibility of these germs to antibiotics [11,12]. It has been reported that up to 25% of cases are treated with the incorrect antibiotic, and in such cases the mortality raises [13,14,15].

Antimicrobial resistance carries a heavy social and economic burden. It is estimated to be responsible for 25,000 deaths per year in the European Union, and 700,000 deaths per year worldwide [16]. By 2050, it is predicted that antimicrobial resistance could cause more deaths than cancer. These circumstances not only involve human suffering, but also serious economic problems [17]. It is estimated that, only in the European Union, antimicrobial resistance costs 1.5 billion euros per year in health costs and lost productivity [16]. In addition, the World Bank has warned that, by 2050, antimicrobial resistant infections could cause economic damage comparable to the financial crisis of 2008 [18].

According to the World Health Organization, antimicrobial resistance has reached alarming levels [19]. Greater efforts are needed to better understand the epidemiology, emergence, prevalence and burden of infectious diseases, in order to improve early detection and better understand antimicrobial resistance challenges in healthcare. Current research in classification of antimicrobial resistance is mainly focused on the use of whole genome sequencing [20,21]. However the whole genome sequencing has a snag, its implementation is more complicated and expensive than other techniques for detecting antimicrobial resistance and it requires waiting for DNA sequencing. A cheaper and simpler method is the culture-based antimicrobial susceptibility testing (CAST), a technique used to determine antimicrobial resistance. CAST is also the main technique employed by clinical laboratories [22]. According to this line of thought, the Kirby–Bauer test [23] is one of the classic and standardized techniques commonly used for determining antimicrobial resistance. The standard workflow to study whether a patient has developed a resistant bacteria based on this test is as follows:

1. A clinical sample is taken from the patient (these samples may be of a different nature, e.g., blood, urine, etc.).
2. A culture is performed on the previously subtracted clinical sample. The goal of the culture is to increase the number of microorganisms, such as bacteria, by preparing an optimal way to promote their development. It is used because many bacterial species are so morphologically similar that it is impossible to differentiate them only with the use of the microscope. In this case, in order to identify each type of microorganism, their biochemical characteristics are studied by planting them in special culture media. The result of a culture will be positive if the microorganism is correctly identified, and negative otherwise.
3. If the culture is positive, the next step is to perform an antibiogram with a determined set of antimicrobials. The antibiogram is constructed from susceptibility testing data and defines the in vitro activity of an antibiotic against a given bacterium (previously isolated in the culture). The antibiogram reflects its ability to inhibit the growth of a bacterium or bacterial population.

The result of the antibiogram usually takes between 24/48 h. Once they have been obtained, resistance and susceptibility are set according to the minimum inhibitory concentration (MIC) of an antibiotic against a microorganism [24]. MIC is defined as the lowest antimicrobial concentration that will inhibit the visible growth of a microorganism after a period of incubation. MIC is obtained from the Clinical Laboratory Standards Institute, and it is periodically updated [24,25]. On the one hand, a bacterium is considered susceptible to an antimicrobial if the antimicrobial is able to create a toxic (usually damage or weakening) effect on the bacterium. On the other hand, a bacterium is considered resistant if it is hardly affected by the application of the antimicrobial. In other words, the antibiogram informs us of the efficacy of the antimicrobials against the bacteria studied.

From a clinical viewpoint, an accurate identification of antimicrobial resistance previous to the result provided by the antibiogram would reduce the time to take important actions such as isolate the patient. In line with this goal, machine learning (ML) techniques [26,27] could be used to create a clinical decision support system to help physicians in this identification. ML has been used in the literature to design systems learning from data, i.e., without establishing a mathematical model in advance [28,29]. This approach has advantages (e.g., flexibility of the designed model), but also limitations (e.g., the quality of the model depends on the data quality and their representativeness). The review of the scientific literature shows the promising results of the use of ML techniques in the healthcare domain [30], and specifically in the analysis of antimicrobial resistance [20,21,31,32,33]. Some of these works are based on genome-sequencing data, which require time for getting the DNA sequencing [20,21]. As a different approach, in this work we propose to predict antimicrobial resistance based on data available in the hospital information systems, which is an important advantage when time is a critical factor.

Specifically, we analyze past data recorded at the ICU of University Hospital of Fuenlabrada (UHF) in Madrid, Spain. We consider demographic and clinical features or variables such as the age, the gender or the mortality risk, among other; variables related to the bacterial culture, for example, the type of sample; and also variables related to the antibiogram, as the antibiotic tested in antimicrobial susceptibility test. Note that, when using a model to predict resistance, the predictor will not require the result of the antibiogram since it is the variable to be predicted. Some features of this dataset have been already analyzed with different purposes by authors of this work. Firstly, ML techniques were used to predict the probability of acquiring nosocomial infection at the ICU [31]. These infections are becoming more relevant since the resistance of some types of bacteria to certain families of antibiotics is increasing. This has been recently shown in [32] using data recorded at the same ICU. Furthermore, authors in [33] analyzed the resistance of different antimicrobial families based on CAST to some frequent bacteria at the ICU based on a visual technique which allows finding linear relationships. Despite being an exploratory analysis technique, it provides useful knowledge to find hidden patterns in data.

Taking into account the above considerations and the previous results, the goal of this paper is to identify new patterns and extract knowledge from them. In particular, we are interested in designing a system able to determine the result (susceptible/resistance) of the antibiogram with just a reduced number of variables. In the literature, these systems are named binary classifiers because the number of potential results is limited to two values. An appropriate design of the classifier would speed up the workflow of the clinical centre to identify and isolate patients potentially in risk of infection.

Therefore, we propose to analyze data available in the health information systems of the ICU of UHF by using ML techniques to detect the emergence of resistance, and to determine the prevalence of resistant bacteria. The goal is to advance in preventive measures, isolations, and achieve a reduction in the rates of emergence of resistant bacteria. In the clinical practice in the ICU of UFH, there are some types of bacteria resistant to certain antibiotics or families of antibiotics with special relevance for their virulence or the possibility to become multidrug-resistant bacteria: *Acinetobacter* spp.; *Enterococcous fecalis* and *Enterococcus faecium*; *Escherichia coli*; *Klebsiella pneumoniae*; *Pseudomonas aeruginosa*; and *Staphylococcus aureus*. In the present study, we focus on *P. aeruginosa* because it is of special relevance in critically ill patients due to its high severity and mortality. The purpose of this paper is twofold:

- To carry out a statistical analysis showing in a map the relationship between certain bacteria and families of antibiotics of special clinical interest.
- To design a ML classifier to determine the resistance of the *P. aeruginosa* bacterium to certain families of antibiotics. Taking into account that the result of the antibiogram usually takes 24/48 h, the use of a data-driven system could help to identify and isolate patients in risk of antimicrobial resistance. From a data analysis viewpoint, we will check which ML scheme provides better performance in terms of accuracy, sensitivity, specificity and F1-score.

The rest of the manuscript is organized as follows. Section 2 presents the notation and the statistical approaches used in this paper. In Section 3 we describe the data set, while results are shown in Section 4. Finally, main conclusions and discussion are drawn in Section 5.

## 2. Statistical Approaches

Machine learning encompasses statistical techniques allowing us to create models by learning the underlying relationships among data [27,28]. In contrast to classical models used for tackling a task, which are defined by humans according to the context knowledge and experience, ML approaches allow us to construct models in a very flexible way. There is a plethora of ML approaches depending on the final goal: interpretability, accuracy, computational cost, etc. When data are not enough representative of the task to solve, the same ML technique can provide different results depending on the data used for learning.

In ML, the characterization of the task is carried out from a set of *N* observations (also called samples), each one composed by *d* elements or features. Thus, the *n*-th sample is defined by a vector $x_{n}=\left[x_{n,1},x_{n,2},x_{n,3}\cdots x_{n,d}\right]$. When learning was performed to find the model relating the input x and the output of a system, it is also necessary to provide the target *y* (desired output). Thus, learning was performed from the set of *N* pairs (input sample, target output):

(1) $$D={\left\{\left(x_{n},y_{n}\right)\right\}}_{n=1}^{N},$$

where $y_{n}$ is the desired output for the input sample $x_{n}$.

According to the purpose of this paper, we present in this section the basis for analysis and identification of antimicrobial resistance. First, we review the Correspondence Analysis (CA) approach, a conventional descriptive technique to summarize the association among categorical features in a visual way. The following subsection presents several ML approaches considered in this work, justifying its choice. The third subsection emphasizes the problem of feature selection to design classifiers with good performance, especially when *d* is high and the number of samples is limited.

### 2.1. Correspondence Analysis

Visualization techniques are useful to understand data and find hidden patterns. In statistics and ML, exploratory data analysis is a crucial step to summarize the main characteristics of a data set, often using visual methods. As an example, histograms, bar diagrams and box plots provide a visual way to analyze each feature individually, allowing us to identify outliers and missing values too. When more than a feature is considered, other tools such as scatter plots are useful when the nature of the features is numerical. However, it is not applicable for categorical variables, i.e., for features with a limited number of options or values (e.g., the categories for the variable ‘eye color’ can be blue, green and brown -one variable, three categories-).

Correspondence analysis [34] is a statistical technique used to analyze, from a graphical point of view, the association among categories of categorical variables. Simple CA considers two categorical variables (otherwise multiple CA is performed) and it projects each category of the variable as a point on a CA map. The total number of created dimensions is the minimum number of categories minus one. The CA map is usually created with two dimensions for interpretation. The proximity between points in the CA map reflects the association between categories.

The simple CA parts of what is known as a contingency table (CT). The CT columns correspond to the categories of one feature, and the CT rows correspond to the categories of the another feature. At the intersection of each row *i* and each column *j*, the absolute frequency $n_{ij}$ (number of elements of the data source) of each combination of categories is stored. Then, the matrix of relative frequencies was computed as $P=\{\frac{n_{ij}}{N}\}$, where *N* is the total number of elements. Two marginal vectors (also named profiles) were generated from P: marginal row (r=P1), and marginal column ($c=P^{T}1$), where ${\left(.\right)}^{T}$ denotes the transpose operator and 1 is a column vector of ones of the proper dimension. Since these profiles are placed in high-dimensional spaces (as many dimensions as number of categories), it is possible to determine subspaces of lower dimension where categories are projected (correspondence map). To obtain coordinates of the row and column, the singular value decomposition (SVD) is used. First, we compute $D_{r}={\left[diag\left(r\right)\right]}^{-1/2}$ and $D_{c}={\left[diag\left(c\right)\right]}^{-1/2}$ to obtain the singular value decomposition (SVD) of the scaled matrix $S=D_{r}{\mathrm{PD}}_{c}$:

(2) $$<U,D_{\alpha },W>=SVD\left(S\right),$$

where the diagonal entries of $D_{\alpha }$ are the singular values of S, matrix W contains the left singular vectors of S, and matrix W the right ones. This allowed us to compute the coordinates matrices (which contain the row and column coordinates) $C_{x}=D_{r}{\mathrm{UD}}_{\alpha }$ and $C_{y}=D_{c}{\mathrm{WD}}_{\alpha }^{T}$. Eigenvalues are computed as the square of the singular values of S, and their sum is named total inertia. In this context, the concept of inertia is equivalent to the concept of variance (variability). The quality of the representation in the correspondence map is measured in percentages of inertia with respect to the total inertia, which is obtained as $\sum_{i}\sum_{j}\frac{{\left(p_{ij}-r_{i}c_{j}\right)}^{2}}{r_{i}c_{j}}$, where $p_{ij}$ is the element in the row *i* and column *j* of P. Dimension 1 represents the largest amount of explained inertia; dimension 2, the second largest and so on.

To analyze the association between the row and column categories, the chi-square distance can be computed as:

(3) $$\chi_{ij}^{2}=\frac{{\left(n_{ij}-e_{ij}\right)}^{2}}{e_{ij}}$$

where $e_{ij}$ are the expected frequencies, computed as

(4) $$e_{ij}=r_{i}c_{j}\frac{1}{N}$$

In general, the higher the chi-square distance is, the stronger the association between the row and column categories. However, it should be taken into account that the chi-square distance considers the marginal proportion of the categories, giving more emphasis to categories with lower frequency of occurrence [35].

The CA analysis seeks: (1) to identify the points (categories) that most contribute to the inertia of each dimension in the map; (2) to identify the points best explained by a factor or dimension (relative contributions). The higher the relative contribution, the better represented is the category in the factor. Regarding the correspondence map, it allows to analyze the association between the categories of each feature separately, and also similarities between the categories of one feature with respect to the categories of the another one. The proximity between categories of different features was interpreted in terms of association.

### 2.2. Machine Learning Techniques

As previously mentioned, there is a variety of ML techniques to identify the susceptibility of a bacteria to certain families of antibiotics. Before applying ML techniques, it is convenient to pre-process data so that these techniques can take advantage of the information in data. Specifically, in this paper we will explore five approaches: logistic regression (LR), voting *k*-nearest neighbors (*k*-nn), decision trees (DT), random forest (RF) and multi-layer perceptron (MLP). LR is a parametric approach commonly used as a baseline because it is a linear classifier. In this work, it will be used to determine the potential of the non-linear classifiers. The other four schemes are non-parametric approaches that can provide non-linear boundaries, i.e., no analytic expression is established for the boundary before training.

The classifier named voting *k*-nn is one of the simplest ML techniques. We consider it because it usually offers good results, since the error rate provided by this technique tends to the minimal one (Bayes error) when *N* is large enough. A bit more complex to design, but also quite interpretable are DT, where nodes of the tree code the rules to perform classification. The simplest DT divide the *d*-dimensional space in a recursive way by considering one feature per node. The performance of DT can be improved by considering an ensemble of DT in the RF scheme. Finally, the MLP moves away from the interpretability and potentially approaches a universal classifier, i.e., a classifier which potentially can model any arbitrary non-linear mapping using just one layer of non-linear neurons.

The best approach to solve a task depends on the data and their characterization, and its selection is done in terms of generalization capacity. It indicates the capacity of the classifier to provide a reasonable output when classifying samples not used for learning. To estimate and compare the generalization capabilities of different classifiers, it is important to separate the set of *N* available samples into two independent subsets, named training set and test set. The training set is used to build the model (classifier) through a learning process (i.e., to find the mapping function). The test set is used to evaluate the performance of the built model. There are different strategies for creating the training and test sets from D. Since a fairly widespread criterion is to randomly assign two-thirds of samples of D to the training set and the rest to the test set, we will follow this criterion. To avoid bias considering just one random partition, it is usual to repeat the train-test partition several times, evaluating each classifier with the corresponding test set. In this work, we have performed 20 random partitions of the train-test sets, providing performance on the test sets.

#### 2.2.1. Data Pre-Processing

There were several challenges associated with the analysis of data collected from the hospital information systems which made some pre-proccesing stages convenient. We next present three of these challenges.

The first challenge was the limitation in the number of samples and the class imbalance [36]. This means that, in general, the number of patients was limited and the prevalence of one class (control) is much higher than the another one (cases). However, most ML techniques provided better generalization when the number of samples was similar for both classes. To solve this issue, several strategies have been proposed in the literature [37]. In this paper, we followed an undersampling strategy [38] which aims to reduce the number of majority samples according to the minority class. More precisely, for each family of antibiotics we undersample the majority class following a simple random sampling (1 to 1) criteria. Then, two-thirds of the samples were considered for training and the rest for testing. As a consequence, the test set is also balanced. The second challenge is related with high dimensional data when the number of patients is limited. In this scenario, feature selection [39] is an important step when building a classifier. The third challenge we discuss in this work is the variety of data types. Health data are usually coded by a large number of both categorical and numerical features, making it difficult to directly run some ML techniques. Therefore, one common strategy is to convert categorical features into binary features using One-Hot-Encoding [40]. One-Hot-Encoding converts any categorical feature into as many binary features as categories there are. All of the new features are encoded by ‘0’ excepting the one mapping the category, which is encoded by ‘1’.

#### 2.2.2. Logistic Regression

The LR is a parametric approach estimating the target value as a linear combination of the input features, which is then run to a logistic function. Coefficients of the linear model are found by optimizing a regularized cost function, to prevent the model from overfitting. The cost function considered in this work is the following:

(5) $$\sum_{i=1}^{d}w_{d}^{2}+C\sum_{i=1}^{N}log\left(exp(-y_{i}\left(x_{i}w^{T}+b\right)+1)\right),$$

where $w=[w_{1},w_{2},w_{3}\cdots w_{d}]$ and *b* are the coefficients to be determined. The first term in Equation (5) is a regularization term (Ridge regularization); the second term is weighted by parameter C>0, which is named the penalty coefficient. The best value for *C* is usually found by exploring a range of values and selecting it by cross-validation. Note that Equation (5) requires that the desired output is coded as {−1,1}.

LR has been commonly used in the literature of health data because it implements a linear classifier, which is the less complex model since the boundary is a hyperplane separating the feature space in two regions.

#### 2.2.3. Voting *k*-nn

The classifiers based on nearest neighbors have their fundamentals on the plausibility that samples close in the representation space have a similar *a posteriori* probability [41]. Proximity among samples is measured by a distance function. In this paper we have considered the Euclidean distance because its widespread use in literature.

Voting *k*-nn classifies a sample z as belonging to the majority class among the *k* nearest samples in the training set. Thus, the closer the samples are in the *d*-dimensional feature space, the smaller the region encompassing *k* samples. Since just a region around a sample is considered for classification, *k*-nn is considered to be a local classifier. The size of the local region around a sample is determined by parameter *k*. In local classifiers, changes in the distribution of samples in a region of the feature space do not affect to the classification in another region of the feature space. This is true for the *k*-nn classifier provided the same *k* value is considered.

For a given a training set, the most complex boundary is built when k=1. In general, when *k* increases, the boundary is getting smoother since the area considered also increases and comes a time that it does not take into account a local area. Note that the appropriate choice of *k* depends on the particular task, and in particular on the number of features, number of training samples and their distribution in the feature space. Best value of *k* is usually found by cross-validation on a range of values.

#### 2.2.4. Decision Trees

Decision trees are classifiers which can be graphically represented in a tree shape as a hierarchical structure [42] starting from a root node. The tree is constructed by splitting nodes into branches in a recursive way according to criteria related to entropy. In this work we have considered the classification and regression tree (CART) [43] because it has been very used in the literature and can manage with heterogeneous features (numerical and categorical) as those characterizing health information. The Gini index, which provides numerical values quite close to the entropy [29], is the criterion used by the CART scheme to select the feature creating new branches in the tree. Every time a node is created, the associated region in the feature space is split in two parts by a linear boundary. A label is assigned to each part according to the majority class in the training samples. Since different regions in the feature space are split in a different way, the whole boundary can be non-linear.

Part of the interpretability of DT relies on the fact that the most discriminative features are closest to the root node, what implicitly could be considered as a feature selection process. Note that the feature selection (FS) process based on mutual information (MI) and also presented in Section 2.3 takes into account the whole training set, while DT perform the feature selection on different regions of the feature space, each one with a different number of training samples.

Since DT are prone to overfitting to the training samples when the number of branches is large, it is convenient to control the tree growth to reduce complexity and get a good generalization capability. Another way to avoid overfitting in DT is to combine the results provided by several DT aiming to solve the same task. This idea leads naturally to the following approach.

#### 2.2.5. Random Forest

The RF approach is based on an ensemble of DT which output is considered to classify a sample according to a majority voting strategy [44]. That is, the RF approach takes the majority class provided by all the individual DT. To improve performance of RF regarding a single DT, it is necessary that trees in the ensemble are diverse. On the one hand, to get this diversity, the training set for designing each DT is created by applying bootstrap with replacement. On the other hand, features considered for splitting each node are not selected from the whole set of *d* features, but from a subset of *m* features (m<d is randomly chosen, usually $m=\sqrt{d}$). Note that RF is closest to a black box model than to an interpretable one. As in individual DT, RF is performed considering the CART algorithm.

#### 2.2.6. Multi-Layer Perceptron

Fundamentals of the MLP are completely different to those from the classifiers presented in previous subsections. On the one hand, the MLP is a global classifier; on the other hand, interpretation of the rules used to define the boundary is really difficult.

The MLP is a kind of artificial neural network organized in layers. It is known that the MLP is a universal classifier even when just one hidden layer of neurons [28]. This capability comes from the fact that neurons implement a nonlinear activation function. Connections between neurons (named weights) are modified during the learning process, which is performed by optimizing a cost function by gradient-based approaches.

The squared error and the cross entropy are among the cost functions most used for MLP training. In this work we consider the cross entropy because of its advantages over the squared error [45,46]. To avoid overfitting, training was regularised by weight decay. Thus, a penalty term (L2 regularization) was added to the cost function to constrain the size of the weights and avoid too much flexibility in the boundary. For optimizing the cost function, we considered the Adam algorithm since it has shown to be computationally efficient and well-suited to a wide range of ML tasks [47].

It is important to remark that the best architecture depends on the particular task. Though there are not established rules to set the MLP architecture, a rule of thumb is that there should be at least 10 samples per weight. A conventional approach (used in this paper) is to explore a wide range of layers and neurons per layer and select the best architecture by cross-validation.

### 2.3. Entropy Criterion for Feature Selection

FS is an essential step to succeed in the building of the ML models. In general, it is convenient to eliminate from vector x in Equation (1) those features that can be noisy, irrelevant or redundant, since they can affect negatively to the proper design of the model. In addition, selecting the most relevant features may increase knowledge and facilitate the interpretation of the final model. Furthermore, a reduced number of features also reduces the model complexity and makes the training process faster.

Feature selection algorithms are usually grouped in three classes according with their interaction with the learning process: filter, wrapper and embedded methods [48]. In filter methods, the selection procedure is carried out independently of the construction of the model. This can be interpreted as a filtering of the irrelevant and redundant variables. Some of these methods are based on entropy, on the coefficient of correlation or on the ‘chi-squared test’ [48,49]. Wrapper methods, such as recursive feature elimination [50], evaluate a subset of attributes based on the performance of the model on a training set. Finally, in embedded methods, the feature selection is done simultaneously with the construction of the model. One of the most common ones is the regularization approach, which offers spare solutions [51].

In this paper, we focus on filter methods due to its simplicity and efficiency. Note that, even when filter methods provide the relevance of each feature without considering the redundancy or the relevance among features, we can benchmark results provided by the filter method with those provided when using features selected by clinicians. Therefore, the FS will be carried out according to two criteria: (1) manual selection by clinicians; and (2) automatic selection by MI.

To define MI, let us first review the information entropy or entropy of Shannon H(.) of a random variable (r.v.) *X* that takes values x∈X. H(X) is a measurement of information, i.e., measures the uncertainty of a variable and it is related to the probability of occurrence of an event [52]. High value of entropy means that each event of *X* has a similar probability of occurrence, whereas low value implies that the probability of occurrence of each event is different. For a r.v. *X*, the entropy is defined as $H\left(X\right)=-\sum_{x\in X}p\left(x\right)log\left(p\left(x\right)\right)$, where p(x) is Pr{X=x}. If another r.v. *Y* that takes values y∈Y is considered, the joint entropy can be computed as $H\left(X,Y\right)=-\sum_{x\in X}\sum_{y\in Y}p\left(x,y\right)log\left(p\left(x,y\right)\right)$, with p(x,y)=Pr{X=x,Y=y}. We can also define the conditional entropy as: $H\left(Y|X\right)\;=\;-\;\sum_{x\in X}\sum_{y\in Y}p\left(x,y\right)log\left(p(y|x\right))$, with p(y|x)=Pr{Y=y|X=x}. The mutual information between *X* and *Y* measures the shared information between them, and is expressed as:

(6) $$I\left(X,Y\right)=\sum_{y\in Y}\sum_{x\in X}p\left(x,y\right)\log\left(\frac{p(x,y)}{p(x)p(y)}\right).$$

We can also define this measure as I(X,Y)=H(X)−H(X|Y)=H(Y)−H(Y|X)=I(Y,X). In other words, MI is the amount of information that the variable *X* has about the variable *Y*. In the context of FS, this can be interpreted as the relevance of the input (independent) feature *X* has with respect to the output (dependent) feature *Y*.

## 3. Data Set Description

The anonymized dataset used in this work was collected from the health information system of UHF for a period of 13 years between 2004 and 2016. During this period, 2630 patients were admitted to the ICU, and 32,997 cultures were carried out in total from 3039 admissions. Note that not all admitted patients have cultures, that the same patient may have been admitted more than once to the ICU, and that several cultures can be performed on the same patient. For each positive culture, different antibiotics were tested to determine if it is susceptible or resistant to the bacteria found in the culture.

We define the term observation as the vector composed of patient/culture/antibiogram data. As an illustrative example, let us consider a patient with 3 cultures and 2 bacteria detected in each culture. If 15 antibiotics are applied in the antimicrobial test, this patient provided a total of 90 observations.

In this work, any observation associated to *Pseudomonas* includes the following sets of features:

- Demographic and clinical features (D&C): age, gender, clinical origin before admission to the ICU, destination after discharge from the ICU, reason for admission, comorbidities, date of admission and date from discharge from the ICU, APACHE II (Acute Physiology and Chronic Health Evaluation, version 2) [53] or SAPS 3 (Simplified Acute Physiology Score, version 3) [54], etc. APACHE II and SAPS 3 are scores used to predict the mortality risk for patients admitted to ICU. APACHE II is performed within 24 h after admission in the ICU and SAPS 3 within one hour. Both of them are related to mortality and severity of illness. Comorbidities are divided in seven groups: Group A (associated with cardiovascular events); group B (kidney failure, arthritis); group C (respiratory problems); group D (pancreatitis, endocrine); group E (epilepsy, dementia); group F (diabetes, arteriosclerosis); and group G (neoplasms). If a patient has more than two comorbidities, the feature named “pluripathology” gets the value 1 assigned.
- Features related to bacterial cultures (BC): the type of clinical sample used in the test (i.e., throat, urine, sputum, feces, wound, etc.), the date on which the culture was carried out and the bacteria found in the culture (if detected).
- Features related to the antibiograms (AT). If the culture is positive, an antibiogram is carry out, which includes: the set of antibiotics tested for each bacteria detected in the culture, their result (susceptibility or resistance) and the date on which the results were obtained, among others.

Table 1 shows a detailed description of the features used in this work to train the ML schemes. The first column identifies the type of feature (D&C, BC or AT) and its name. If the feature was numerical, the information related to the category (second column) and subcategory (third column) does not apply. The only exception was the feature named “culture date”, which was divided into “year of culture”, “month of the culture” and “day of the culture” to be analyzed in an easier way. If the feature was categorical, we describe its categories or subcategories in the second and third columns, respectively. For the ML schemes, each categorical feature is replaced by a new set of features, as many as categories or subcategories. The column named “# feat.” in Table 1 indicates the number of considered features for FS and ML purposes hereinafter. For example, the original feature “reason for admission” has 7 categories (surgery, respiratory, cardiovascular, infection, other medical, neurology and trauma), each of them with subcategories. The category surgery had the following four subcategories: “surgery scheduled with complications”, “surgery scheduled without complications”, “urgent surgery with complications”, and “urgent surgery without complications”. The category respiratory had three subcategories, the category cardiovascular had six, infection had two, other medical had six, neurology had five, and trauma had one. Therefore, the original feature “reason for admission” is coded into 27 features, which are considered for the FS and the ML techniques. The column on the right of Table 1 presents the mean±standard deviation value for numerical variables; for categorical variables, the value represents the percentage of observations in each category.

**Figure 1:**
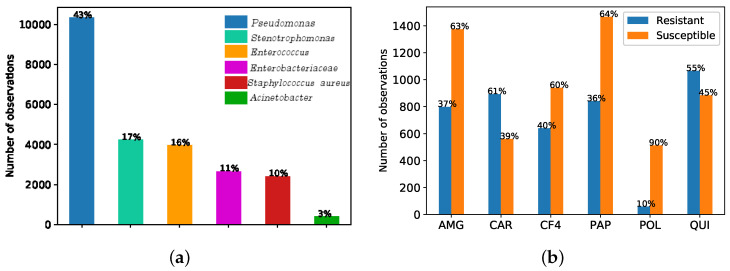
(**a**) Bar graph of number of observations per germen when considering six antimicrobial families of interest (aminoglycosides (AMG), carbapenemics (CAR), fourth-generation cephalosporins (CF4), broad-spectrum antibiotics (PAP), polymixines (POL), and quinolones (QUI)). (**b**) Bar graph of number of observations of the most frequent germen (*Pseudomonas*) in terms of resistant and susceptibility for each antimicrobial family.

The records considered in this work provided a total of *N* = 144,475 observations. According to the notation in Equation (1), *N* is the total number of observations, x is the feature vector (see Table 1 for a description of the considered features), and the target *y* is a binary variable encoding the result (susceptible/resistant) of the antibiogram to the kind of bacterium using MIC, which is obtained from the Clinical Laboratory Standards Institute as explained in Section 1. These features are used to identify antimicrobial bacteria resistant to *Pseudomonas*. Figure 1a shows that the most frequent germen is *Pseudonomas*, representing the 43%, followed by *Stenotrophomonas* (17%), *Enterococcus* (16%), *Enterobacteriaceae* (11%), *Staphilococcus aureus* (10%) and the *Acinetobacter* (with just the 3% of the bacteria present in the ICU at UHF). Figure 1b shows the observation rate associated to resistance (susceptibility) to *Pseudonomas* for each antimicrobial family of interest, namely, aminoglycosides (AMG), carbapenemics (CAR), fourth-generation cephalosporins (CF4), broad-spectrum antibiotics (PAP), polymixines (POL), and quinolones (QUI).

## 4. Results

### 4.1. Visualization Based on CA

We analyzed here the CA map to find the association between bacteria and families of antibiotics. Figure 1a shows the six more frequent types of bacteria when considering the six antimicrobial families of interest. According to the antibiogram results (see Figure 1b) and clinical knowledge, *Pseudomonas* have shown high resistance to CAR.

In this work, we used CA as a visual technique to understand clinical associations among the more frequent bacteria and different families of antibiotics. We focused on the three most frequent bacterial types to represent the total inertia in just two dimensions (note that the total number of created dimensions is the minimum number of categories minus one). These bacteria were considered to evaluate their resistance to three families of antibiotics, namely, AMG, CAR and QUI, since they are also the most frequent for the considered bacteria. Towards that end, the CT for the two variables: kind of bacteria (3 categories) and antimicrobial family (3 categories) was built. It has been accomplished by counting the number of observations such that certain bacteria are resistant to certain family of antibiotics (see Table 2 for details). *Pseudomonas* and *Enterococcus* exhibit the highest occurrence for the QUI, in opposition to *Stenotrophomonas*, which presents the lowest ocurrence.

**Figure 2:**
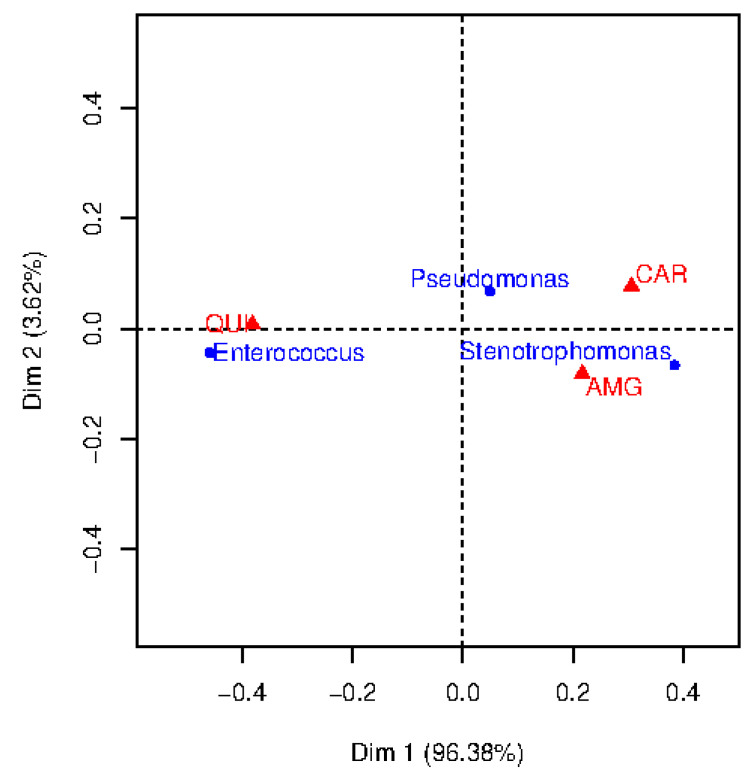
Correspondence analysis map of bacterial type and antimicrobial family when considering resistant observations.

A bi-dimensional correspondence map for resistant observations is shown in Figure 2. To determine the number of dimensions considered in the CA map, it is usually recommended to retain a number of dimensions representing more than 70% of the inertia. Though we decided to consider two dimensions for a better visualization, note that Dimension 1 (horizontal axis) provides much more inertia (96.38%) than Dimension 2 (vertical axis, 3.62%). The points in this map represent the categories of each feature (represented by triangles, the categories associated to the family of antibiotics; represented by circles, the categories associated to the bacterial type). To interpret the CA map, the origin on the map (coordinates (0,0)) corresponds to the centroid of each variable. Thus, the further away is the point from the origin along a particular dimension, the greater its importance on that dimension. The position of the point in the map can indicate associations between bacteria and family of antibiotics.

Chi-square distances were evaluated to analyze the resistance between each germen and each family of antibiotics (see Table 3 for details). In general, the higher the chi-square distances are, the stronger the association. According to this, several clinical conclusions can be drawn. For example, *Pseudomonas* show high resistance to CAR (chi-square distance 13.8), whereas *Enterococcus* is resistant to QUI (chi-square distance 208.8). For *Stenotrophomonas*, the chi-square distance is high for QUI, however, this is not reflected in the two-dimensional map. The reason is that the chi-square distance is emphasizing this category since it has low frequency of occurrence (see Table 2).

Since CA is an exploratory method to analyze relations among categories and has no predictive power, we explore the use of machine learning techniques in the rest of the paper.

### 4.2. Antimicrobial Resistance Identification

This section starts by discussing the experimental set-up and describing the built models with emphasis on the process to train them. We subsequently analyzed the FS approaches considered in this work. Finally, different ML techniques are evaluated when considering the two FS strategies, showing that results improve significantly when considering a criterion based on MI.

#### 4.2.1. Experimental Set-Up

Figure 1a shows that the most relevant bacteria at the ICU of UHF are *Pseudomonas*, and for that reason, as already mentioned, we focused on this bacterium. A total of 10,048 observations associated to the antibiograms done for positive cultures of *Pseudomonas* were evaluated. Since the performance in terms of susceptibility and resistance of the *Pseudomonas* to different families of antibiotics can be different, six classifiers were designed:

- Antimicrobial family 1. Aminoglycosides (AMG).
- Antimicrobial family 2. Carbapenemics (CAR).
- Antimicrobial family 3. 4${}^{\circ }$G Cephalosporins (CF4).
- Antimicrobial family 4. Broad spectrum antibiotics (PAP).
- Antimicrobial family 5. Polymixines (POL).
- Antimicrobial family 6. Quinolones (QUI).

The methodology performed to train and evaluate each model was the following. First, the data set was filtered in order to obtain only the observations corresponding to the bacterium and family of antibiotics of interest. Table 4 shows the total number of observations for each antimicrobial, as well as the number of samples in the minority class (and the percentage), where *R* was used to denote resistance and *S* identifies that the antimicrobial is sensitivity. The family with most observations was PAP (64% R), AMG (36% R), QUI (45% R), CF4 (59% R), CAR (62% R), with POL being the family with the least observations (10% R). Since the observations of interest were imbalanced and ML approaches can be sensitive to it, we followed an undersampling strategy as explained in Section 2.2.1.

After class balancing, the two-thirds of samples were randomly assigned to the training set and the rest to the test set. We repeat this process 50 times so that the provided values for performance are less biased to particular good or bad partitions. Results were provided in terms of mean and standard deviation of the accuracy, sensitivity, specificity and F1-score [55].

#### 4.2.2. Feature Selection

In this section, we turn our attention to the analysis of the proposed FS strategy based on mutual information (**FS2**), and benchmark results with a manual selection approach performed by clinicians (**FS1**). The feature selection based on clinical knowledge was carried out with the help of the clinical staff from the UHF. According to the experience and knowledge of the clinical staff, features related to the year of the culture, antibiotics of minor importance and the clinical units of origin have not been chosen.

The feature selection based on MI was performed for each antimicrobial family considering just the training sets. As it can be shown in Figure 1b, the number of observations for each antimicrobial family is different. After balancing classes according to the minority one, observations in certain families of antibiotic did not have some features listed in Table 1, and therefore they cannot be considered for the FS and ML schemes. This means that the total number of features can be different for each family, being 127 features for family 1, 119 for family 2, 122 for family 3, 126 for family 4, 78 for family 5, and 126 for family 6.

**Figure 3:**
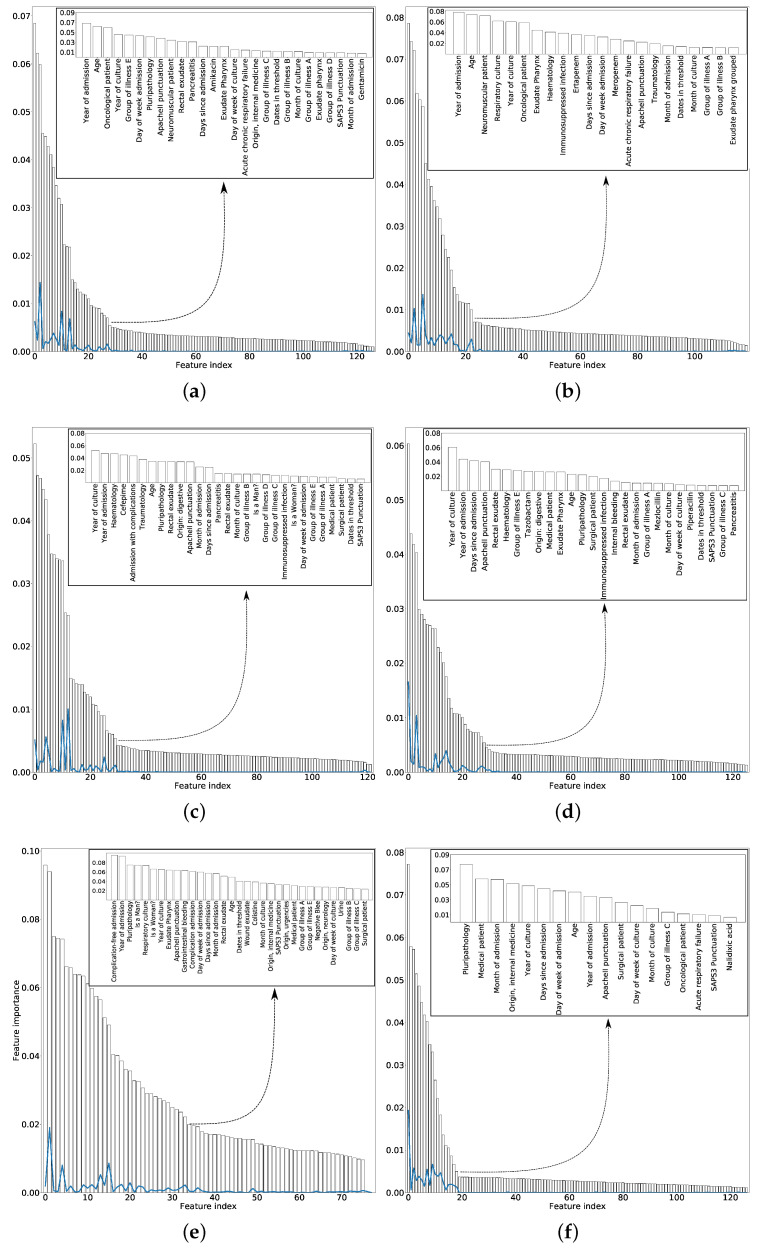
Mean values, ranked in descending order, for the MI between each feature and the target when considering 50 different training sets for: (**a**) family 1. *Pseudomonas*—AMG; (**b**) Family 2. *Pseudomonas*—CAR; (**c**) Family 3. *Pseudomonas*—CF4; (**d**) Family 4. *Pseudomonas*—PAP; (**e**) Family 5. *Pseudomonas*—POL; and (**f**) Family 6. *Pseudomonas*—QUI. The solid lines represent the difference between the importance of a certain feature and the MI of the next one in the figure. The selected features for each family are inset.

For each antimicrobial family, Figure 3 shows the total number of features sorted according to the mean MI obtained for 50 different training sets (randomly selected). Inside the figure, we show the selected features. In order to choose the number of variables that will allow to obtain the optimum information gain, the same stopping criterion has been used for all families of antibiotics. This stopping criterion was based on sorting the variables by importance (see Figure 3) and choosing those features whose importance is high enough and not yet similar to the others features. To do this, the solid lines in Figure 3 have been used. These lines computed the difference between the importance of a certain variable minus the importance of the next. As they were ordered by importance, when the line had values close to 0, it means that the variables had similar importance, therefore, we have selected the last local maximum of these lines.

In almost all the families of antimicrobial, the number of features selected was between 20 and 30, therefore it can be concluded that most of the features (around 120) were irrelevant. The selected features in the case of the POL was larger, possibly due to the fewest number of samples in comparison with the other families. Some features are recurrently selected among the six antimicrobial families, among them: scores used to predict the hospital mortality, such as Apache II or SAPS 3, the age of the patient; the clinical unit the patient comes from (for example, internal medicine or surgery); or temporal features such as the days from admission to the culture.

### 4.3. Classification Results

The goal of this subsection is to predict the susceptibility or the resistance to certain antimicrobial families to the *Pseudomonas*. Towards that end, we considered two FS strategies, FS1 and FS2, described previously. For each feature subset, the following ML classifiers were benchmarked: LR, *k*-NN, DT, RF and MLP. Each classifier depends on different hyperparameters. Values for the hyperparameters were selected as those with the best performance according to the five-fold cross-validation strategy (leaving the test set apart). The hyperparameters for each model are the following:

- LR: penalty coefficient *C* in regularization. For obtaining it, we have considered two grids of values. The first grid explores values logarithmically spaced in the range between 0.01 and 10. A second grid was subsequently used around the best value found.
- *k*-NN: number of neighbors *k*. A range between 1 to 49 was considered, only taking odd values to address ties.
- DT: maximum depth of the tree, from 1 to 35. For the minimum number of samples per leaf, we considered the 0.5%, 1% and 2% of the samples.
- RF: number of trees (estimators) in the forest {10, 30, 50, 100 to 150}. For the maximum depth of each tree and the minimum samples per leaf, the same range of values considered in decision trees has been explored.
- MLP: one and two hidden layers were considered. For the first hidden layer, the number of neurons ranged from 50 to 70; and from 0 (no hidden layer) to 5 in the second hidden layer. Different activation functions have been considered, logistic, tanh and relu. For the L2 penalty coefficient, we have considered two grids of values. The first grid explores values logarithmically spaced in the range between 0.01 and 10. A second grid was subsequently used around the best value found in steps of 0.01.

Table 5 shows the hyperparameters selected for designing each classifier following a cross-validation approach. The performance with these hyperparameters was evaluated in the test set in terms of accuracy, sensitivity, specificity and F1-score. Since we are considering 50 different training/test sets, we provide the mean and the standard deviation on the test subsets (see Table 6).

**Figure 4:**
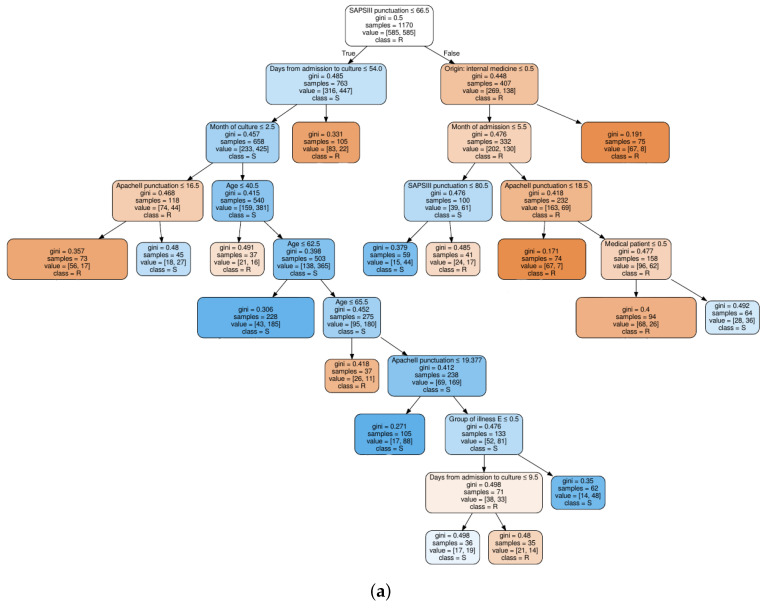

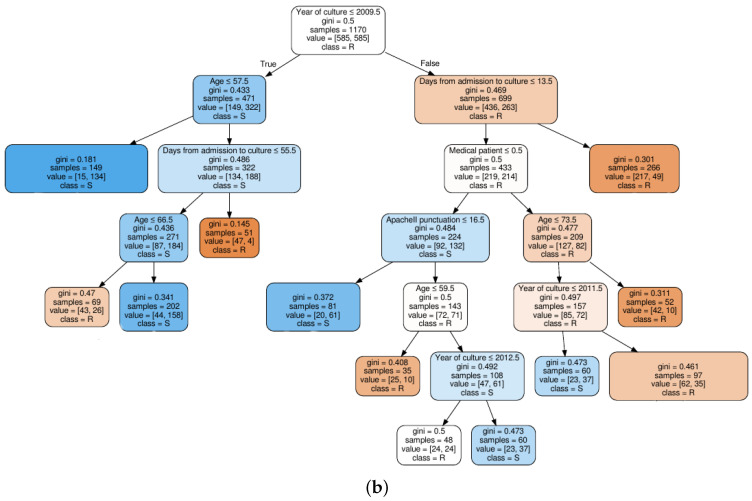
Decision tree classifier trained on quinolones observations: (**a**) based on clinical knowledge feature selection procedure; and (**b**) based on MI feature selection procedure.

Several conclusions can be obtained from Table 6. In general, better performance is obtained when ML models are designed based on FS according to MI (FS2) for all figures of merit. On the other hand, results evidence that non-linear schemes are better to identify antimicrobial resistance at the ICU, with performance depending on the family of antimicrobials. As expected, LR provides the worst results for all families of antibiotics except for POL. In line with this comment, note that all models created for POL show the highest variance in their performance, maybe because of the reduced number of observations for this family (see Figure 4). The high number of features chosen in FS1 in relation to the number of observations may be the cause of the very bad results provided by DT (especially for sensitivity and F1-score). On the other hand, QUI is the antimicrobial family with the highest number of observations (see Figure 4), and it is the family with higher accuracy (90.1), specificity (90.2), sensitivity (90.5) and F1-score (90.0) for the *k*-nn model. It is particularly relevant to emphasize the capacity to identify resistant observations for this antimicrobial family.

The MI feature selection chose the scores APACHE II and SAPS 3 as important features for all families of antibiotics. To examine the potential influence of these features, we trained a *k*-nn classifier in the same way as Table 6 for FS2, but excluding APACHE II and SAPS 3. Note that this model design was against the decision of the feature selection procedure. Results are shown in Table 7, where four performance measurements were considered (accuracy, specificity, sensitivity and F1-score). We compared these results with those in Table 6, concluding that similar performance is obtained when excluding APACHE II and SAPS 3 scores.

For a clinical interpretation of the results, we show also an example of two decision trees trained on the QUI family of antibiotics, when considering the FS1 approach (see Figure 4a), and when considering the FS2 approach (see Figure 4b). Selection of the QUI family was motivated by the good performance of our models for this family of antibiotics. It can be seen that the tree in Figure 4b is much shallower than tree in Figure 4a, since the number of features was smaller.

We can conclude that the age, days from admission to culture, APACHE II and SAPS 3 features appear in several splits of both trees, which means that they are relevant factors to know if a patient will develop a resistant bacteria against the QUI family of antibiotics. Specifically, the feature named year of admission is the most relevant one. This fact allows us to identify a change in the trend of antimicrobial resistance developed by *Pseudomonas* to QUIN from 2009 (see root node). Further away from the root node are features as month of admission, pluripathology and medical patient.

## 5. Discussion and Conclusions

Several studies have underlined the relevance of antimicrobial resistance, since it continues increasing worldwide [56,57]. This is closely related to the difficulties to treat bacterial infections, which could potentially cause harm, damage or even death.

Among the most frequent bacteria, *Pseudomonas* and *Stenotrophomonas* are two families of microorganisms that cause infections of great virulence in patients admitted to the ICU. The severity of the infection is linked to the frequent resistance of these bacteria to many families of antibiotics, making the treatment of infections difficult. The resistance of these microorganisms has been increasing over the years (as shown in [32]), and it is common to find clinical samples that are only susceptible to just a one family of antibiotics.

The antimicrobial resistance conducts to delay in using the appropriate therapy based on the effective treatment, and to use more antibiotics. This fact is specially relevant at the ICU [58], where the administration of prolonged antimicrobial and antibiotic pressure are higher due to the patient health status. Antimicrobial resistance leads to longer stays at the hospital, higher mortality rate and costs of care. Apart from that, and of great interest, it is the necessity to control the spread of antimicrobial resistant bacteria and to identify them in advance to isolate patient as soon as possible. Given all this, there is urgent to propose new methods to identify antimicrobial resistance and acting as quickly as possible. Furthermore, the identification of relevant features could provide knowledge to identify risk factors and reduce the nosocomial infections.

According to internal reports in the ICU, in 2018, the incidence rate of multidrug-resistant bacteria in the Spanish ICUs was eight patients with one or more multiresistant germs per 1000 patient days, or 4.2 patients with one or more resistant germs per 100 patients admitted. Strict control of the mechanisms of cross-infection among patients and an adequate antibiotic policy are essential requirements in the fight against infection by resistant bacteria in the ICU. Recently, several studies have analyzed the genome-sequencing data using machine learning techniques to predict antimicrobial resistance [20,21]. Our work is based on data available at ICUs on the daily clinical practice (clinical and demographic variables from the patient, as well as data from cultures and antibiograms). These data do not require waiting, as it happens for example in DNA sequencing.

In this paper, we propose an strategy based on FS and ML techniques to identify antimicrobial resistance to *Pseudomonas*. On the one hand, the feature selection based on MI has demonstrated that a great number of the features considered in this work were not relevant. It has also been shown how ML methods (i.e, LR, *k*-NN, DT, RF and MLP) could be useful to accelerate the workflow of clinical centers.

The methodology proposed in this work may allow us to anticipate results provided by the microbiology laboratory. The early identification of patients with high risk to be resistant to one or several families of antibiotics may derive useful knowledge for the patient, for the healthcare system and for the society in general. As an immediate advantage, it could help to determine the appropriate antimicrobial therapy. Furthermore, those patients whose results of the cultures have been identified as resistant could be isolated, being able to stop possible outbreaks of resistant bacteria, and therefore, to avoid crossed transmission to other patients in the ICU. This action may be translated into a lower workload, lower mortality and in a reduction of infections during the stays of the patients in the ICU.

Good performance was achieved only taking into account the dates in which the culture was made, the antibiotic supplied, the clinical origin of the patient, the disease which is the reason for admission in the ICU, the time elapsed from the patient was admitted until the culture was performed, APACHE II and SAPS 3 scores. We have examined the potential influence of APACHE II and SAPS 3 scores to identify antimicrobial resistance. Towards that end, we trained a *k*-nn classifier excluding both scores as selected features (going against the decision of the feature selection procedure). Results in terms of different figures of merit revealed similar results as the ones obtained when including APACHE II and SAPS 3 together with the rest of features. So, even when the potential of each of these scores is high when considered alone, this potential decreases when they are considered in combination to other features. In line with these insights, authors in [59] conducted a meta-analysis with 18 publications to identify risk factors for multidrug-resistant Gram-negative bacterial infection in ICUs. Factors such as age, gender, hospital stay, APACHE II score, medication, comorbiditiy, mechanical ventilation, etc., were identified. Among them, a total of six factors were more likely to be associated with multi-drug-resistance: having an operative procedure, a central venous catheter, mechanical ventilation, length of ICU stay.

Future work concerns deeper analysis of other clinical features to extract more knowledge about the bacterial transmission in the ICUs, as well as the identification of potential risk factors. Among these features, we will consider mechanical ventilation, the bed where the patients are, or even variables at ICU level as the number of patients with sepsis or the total number of nurses working in the unit. In line with this idea, we think that it would be of great interest to understand the influence of the use of certain antibiotics can have on the appearance of certain bacteria.

Furthermore, from a methodological viewpoint, new approaches could be considered. For example, different strategies to balance classes based on novel ML approaches such as the generative adversarial networks [60], could be lead to generate patients records instead of considering an undersampling strategy. Apart from that, on the one hand, wrapper or embedded FS; and on the other hand, other ML schemes such as eXtreme gradient boosting (XGBoost) or support vector machine (SVM) could provide better results. We will extend the current study to the identification and prediction of multi-drug resistance instead of just simple resistance as done in this work. Furthermore, since the data analyzed so far ran until 2016, work is currently underway to include new data from 2016 to 2019. These new observations will be used to test the trained models. It would also be interesting to test the models with data from the ICU of other hospitals.

All in all, we can conclude that antimicrobial resistance is an actual problem that is currently facing in medicine. The use of data recorded in the EHR and its analysis by ML techniques could improve clinical practice in the ICU. This would result both in personal benefits (health status of the patient) and socio-economic benefits (management of the health system).

## Figures and Tables

**Table 1:** Data set description.

Type—Feature	Category	Subcategory	# feat.	Mean ± std
**D&C—Age**	-	-	1	62 ± 14
**D&C—SAPS3**	-	-	1	61 ± 14
**D&C—ApacheII**	-	-	1	20 ± 7
**BC—Days from**	-	-	
**adm. to culture**	-	-	1	12.1 ± 18.4
**BC—Culture Date**	Year,	-	3	2010 ± 4,
	Month, Day	-		6 ± 3, 2 ± 2
**Type—Feature**	**Category**	**Subcategory**	**# feat.**	**% of obs.**
**D&C—Gender**	Male Female	-	1 1	61.37 38.63
**D&C—Diagnosis**	Groups	A, B, C, D,	8	14.9, 11.3, 25.1
		E, F, G, pluripathology		7.6,8.8, 25, 28.9
**D&C—Patient type**	-	Surgical, Medical, Trauma	3	100
**D&C—Reason for admission**	Surgery	Scheduled with (without) complications, Urgent with (without) complications	4	25.6
	Respiratory	Chronic acute respiratory insufficiency, respiratory failure, Respiratory other	3	21.2
	Cardiovascular	Heart failure, Ischemic heart disease, Severe arrhythmia, Cardiorespiratory arrest, Hypovolemia, Cardiovascular other	6	16.7
	Infection	Serious infection, Immune-compromised infection	2	16.2
	Other medical	Digestive haemorrhage, Diabetic decompensation, Acute renal failure, Hepatic insufficiency, Voluntary intoxication, Pancreatitis	6	9.7
	Neurology	Stroke, Epilepsy, Alteration of the awareness level, Neuromuscular, Neurological other	5	9.0
	Trauma	Severe trauma	1	0.8
**D&C— Origin**	Emergency	-	1	27.4
	General surgery	-	1	26.7
	Internal medicine	-	1	11.1
	Others	Anesthesia, Dermatology, Digestive, Gastrointestinal, Hematology, Nefrology, Neumology, Neurology, Oncology, Other hospital, Others, Otorrinolaringology, Psychiatry, Surgery, Traumatology, Urology, Ophthalmology	19	34.8
**BC—Type of sample**	Exudate	Rectal, Nasal, Axillary, Pharyngeal, Inguinal, Wound, Urethral, Press ulcer	20	91.9
	Others	Blood, Catheter, Urine, Feces, Abscess, Abdominal abscess, Respiratory, Drainage, Abdominal drainage, Abdominal fluid, Sputum, Pleural, Bronchoalveolar lavage, Secretion, Peritoneal liquid, Ascitic liquid, Biliary liquid	29	8.1
**AT—Antibiotics**	Others	Amikacin, Gentamicin, Gentamicin high load synergy, Kanamycin high load synergy, Tobramycin, Imipenem, Meropenem, Ertapenem, Ceftazidime, Cefepime, Piperacillin, Ticarcillin, Mezlocillin, Colistin, Ciprofloxacin, Levofloxacin, Norfloxacin, Nalidixic acid, Ofloxacin, Moxifloxacin	20	100

**Table 2:** Contingency table used in the correspondence analysis for resistance.

	Fam. Antim.	AMG	CAR	QUI
Bacterial Types				
*Pseudomonas*		802	897	1065
*Stenotrophomonas*		712	633	368
*Enterococcus*		396	250	1085

**Table 3:** Chi-square distances for resistant observations used in correspondence analysis.

	Fam. Antim.	AMG	CAR	QUI
Bacterial Types				
*Pseudomonas*		2.8	13.8	2.8
*Stenotrophomonas*		64.9	41.0	153.7
*Enterococcus*		35.0	122.2	208.8

**Table 4:** Total number of observations (first row) and number of observations in the minority class (second row; in brackets, percentage) for each family of antimicrobians when *Pseudomonas* are considered. Label S in brackets stands for susceptible, and label R for resistant.

	AMG	CAR	CF4	PAP	POL	QUI
**Total observations**	2177	1458	1582	2309	570	1952
**# of observations in the minority class**	802	560	642	842	58	884
**(%)**	(36% R)	(38% S)	(41% S)	(36% S)	(10% R)	(45% R)

**Table 5:** Hyperparameters found when considering a five-fold cross-validation strategy on the training set for each model associated to the feature selection (FS) strategy and the antimicrobial family.

FS	Model	Hyperparameter	AMG	CAR	CF4	PAP	POL	QUI
**FS1**	**LR**	Penalty coefficient	0.73	0.62	0.48	0.13	0.05	0.01
	***k*-nn**	N° neighbors	1	1	1	1	5	1
	**DT**	Max. depth	22	23	31	20	22	20
		Min. samples per leaf	5	4	8	6	5	6
	**RF**	Max. depth	37	25	30	38	14	20
		Min. samples per leaf	5	4	4	6	5	6
		N° of tress	50	100	30	50	50	100
	**MLP**	Activation function	Relu	Relu	Relu	Relu	Relu	Relu
		L2 penalty coefficient	0.02	0.01	0.01	0.01	0.20	0.05
		N° of neurons	59	60	59	62	58	61
**FS2**	**LR**	Penalty coefficient	0.04	1.50	0.12	1.01	0.01	0.05
	***k*-nn**	N° neighbours	1	1	1	1	7	1
	**DT**	Max. depth	22	15	41	19	3	4
		Min. samples per leaf	5	4	5	6	1	20
	**RF**	Max. depth	30	18	18	22	26	30
		Min. samples per leaf	5	4	4	6	4	6
		N° of trees	50	50	50	50	30	30
	**MLP**	Activation function	Relu	Relu	Sigmoid	Relu	Relu	Relu
		L2 penalty coefficient	0.01	0.01	0.10	0.01	0.13	0.03
		N° of neurons	64	64	62	63	59	57

**Table 6:** Mean ± standard deviation of the performance (accuracy, specificity, sensitivity, F1-score) provided by five kind of models (second column) on 50 test sets. The goal is to determine resistance of *Pseudomonas* to six families of antimicrobials (first column). Two FS approaches have been considered: clinical knowledge (FS1) and mutual information (FS2). Bold figures refer to the highest performance per figure of merit and antimicrobial family.

Family	Model	Accuracy		Specitivity		Sensitivity		F1-score	
		FS1	FS2	FS1	FS2	FS1	FS2	FS1	FS2
AMG	LR	78.2 ± 1.2	75.3 ± 1.7	80.0 ± 1.9	77.2 ± 2.7	76.5 ± 1.8	73.5 ± 2.4	77.8 ± 1.2	74.8 ± 1.7
	*k*-nn	79.3 ± 1.6	**83.3 ± 1.9**	84.0 ± 2.5	**86.5 ± 2.2**	74.5 ± 2.1	**80.3 ± 2.0**	78.1 ± 1.3	**82.7 ± 2.0**
	DT	77.0 ± 1.2	78.6 ± 2.3	78.0 ± 3.7	81.7 ± 2.5	75.9 ± 2.6	76.6 ± 2.7	76.5 ± 1.1	78.0 ± 2.6
	RF	80.1 ± 1.6	80.8 ± 1.2	80.0 ± 2.3	81.3 ± 2.4	80.0 ± 2.0	80.2 ± 2.0	80.0 ± 1.6	80.5 ± 1.5
	MLP	80.8 ± 1.3	78.3 ± 1.0	83.0 ± 2.1	82.0 ± 1.7	78.6 ± 1.7	75.0 ± 1.2	80.1 ± 1.3	77.9 ± 1.1
CAR	LR	77.3 ± 1.4	74.8 ± 2.3	76.0 ± 3.0	72.0 ± 3.4	78.7 ± 2.8	77.8 ± 3.1	77.6 ± 1.8	75.5 ± 2.3
	*k*-nn	79.9 ± 1.6	81.5 ± 1.4	80.0 ± 2.8	80.3 ± 2.5	80.1 ± 2.5	82.7 ± 2.2	79.8 ± 1.7	81.6 ± 1.7
	DT	78.1 ± 2.2	79.4 ± 2.0	80.0 ± 2.6	**83.7 ± 3.4**	75.8 ± 2.7	76.2 ± 3.2	77.3 ± 2.7	78.9 ± 2.0
	RF	**82.4 ± 1.7**	82.2 ± 1.7	78.0 ± 4.0	82.0 ± 3.1	**86.8 ± 3.1**	82.5 ± 2.6	**83.2 ± 1.5**	82.5 ± 1.6
	MLP	81.9 ± 1.5	79.0 ± 1.9	81.0 ± 3.3	78.6 ± 2.5	82.6 ± 2.9	80.2 ± 2.1	82.3 ± 1.5	79.2 ± 1.8
CF4	LR	68.7 ± 2.0	67.8 ± 1.3	70.0 ± 3.1	68.1 ± 2.3	67.9 ± 2.6	67.1 ± 2.1	68.2 ± 2.3	67.3 ± 1.8
	*k*-nn	77.7 ± 1.5	75.6 ± 1.6	**80.0 ± 2.7**	78.9 ± 2.6	75.2 ± 2.4	72.9 ± 2.6	77.0 ± 1.4	74.7 ± 2.0
	DT	71.0 ± 1.1	74.6 ± 2.2	74.4 ± 3.7	78.0 ± 3.6	67.9 ± 3.2	71.8 ± 3.1	70.3 ± 1.4	73.9 ± 2.4
	RF	75.8 ± 1.9	**78.0 ± 1.8**	72.0 ± 4.3	77.2 ± 3.7	79.4 ± 3.0	**79.6 ± 3.3**	76.7 ± 1.7	**78.2 ± 1.8**
	MLP	77.0 ± 1.5	75.8 ± 1.4	81.0 ± 4.5	77.4 ± 3.6	73.4 ± 3.7	75.1 ± 2.7	76.6 ± 1.1	75.3 ± 1.9
PAP	LR	69.0 ± 1.4	67.7 ± 1.4	68.0 ± 2.8	65.9 ± 3.0	70.4 ± 2.5	70.2 ± 2.5	69.1 ± 1.6	68.1 ± 1.4
	*k*-nn	78.3 ± 1.6	**78.4 ± 1.4**	**82.0 ± 2.5**	81.3 ± 2.2	74.8 ± 2.4	76.3 ± 2.7	77.4 ± 1.9	77.7 ± 1.7
	DT	72.6 ± 2.2	74.6 ± 2.0	74.0 ± 3.5	78.1 ± 4.1	70.9 ± 2.6	71.1 ± 3.5	71.7 ± 2.5	73.4 ± 2.3
	RF	75.2 ± 1.5	75.6 ± 1.2	71.0 ± 3.0	73.0 ± 3.0	**79.5 ± 2.7**	78.3 ± 2.4	75.7 ± 1.8	76.1 ± 1.4
	MLP	78.2 ± 1.7	75.4 ± 1.4	78.0 ± 1.8	76.3 ± 2.3	78.0 ± 3.3	74.4 ± 1.9	**78.1 ± 2.4**	75.1 ± 1.8
POL	LR	68.1 ± 6.3	70.95 ± 6.8	63.0 ± 7.7	65.1 ± 10.0	73.4 ± 6.3	**76.3 ± 8.4**	69.6 ± 6.6	71.4 ± 6.9
	*k*-nn	63.9 ± 7.2	70.5 ± 7.0	68.0 ± 12.5	74.0 ± 6.7	61.4 ± 11.8	67.4 ± 7.4	63.0 ± 9.3	69.2 ± 9.0
	DT	60.6 ± 5.2	**72.1 ± 6.9**	65.0 ± 12.2	69.3 ± 14.6	57.0 ± 11.6	75.5 ± 7.9	58.3 ± 7.8	**71.5 ± 7.0**
	RF	65.3 ± 7.9	68.9 ± 6.9	58.0 ± 15.0	66.4 ± 11.2	74.2 ± 11.2	73.4 ± 6.6	68.4 ± 6.9	70.0 ± 6.2
	MLP	67.7 ± 3.5	70.8 ± 6.2	**75.0 ± 7.6**	71.0 ± 12.4	60.5 ± 9.2	71.5 ± 9.5	64.4 ± 2.3	71.3 ± 5.8
QUI	LR	72.1 ± 2.1	71.8 ± 1.5	70.0 ± 2.8	71.6 ± 2.4	74.2 ± 2.5	72.2 ± 2.6	72.7 ± 2.1	71.9 ± 1.9
	*k*-nn	86.8 ± 1.1	**90.1 ± 1.3**	86.0 ± 1.6	**90.2 ± 1.9**	87.6 ± 1.7	**90.5 ± 1.7**	86.8 ± 1.3	**90.0 ± 1.4**
	DT	81.8 ± 1.7	82.3 ± 1.5	83.7 ± 2.8	85.0 ± 2.8	80.1 ± 2.2	79.6 ± 2.2	81.4 ± 2.1	81.7 ± 1.7
	RF	82.5 ± 1.8	83.6 ± 1.4	79.0 ± 2.9	84.6 ± 2.4	86.0 ± 2.9	83.6 ± 2.0	82.9 ± 1.7	83.7 ± 1.3
	MLP	87.1 ± 1.4	83.4 ± 2.0	87.0 ± 0.8	82.7 ± 1.8	87.0 ± 1.0	84.8 ± 1.7	86.7 ± 1.7	83.7 ± 2.0

**Table 7:** Mean ± standard deviation of the performance (accuracy, specificity, sensitivity, F1-score) provided by the *k*-nn model on 50 test sets. Results are shown for every antimicrobial family (first column) after excluding the scores APACHE II and SAPS 3 from the set of selected features by MI.

Antimicr. Family	Accuracy	Specificity	Sensitivity	F1-Score
AMG	82.2 ± 1.7	86.0 ± 2.3	78.7 ± 2.3	81.6 ± 1.9
CAR	79.6 ± 2.1	81.0 ± 3.5	78.3 ± 2.8	79.0 ± 2.0
CF4	74.9 ± 2.1	77.0 ± 3.7	72.6 ± 2.6	74.3 ± 2.0
PAP	77.1 ± 1.7	80.0 ± 2.9	74.0 ± 2.5	76.1 ± 1.8
POL	68.5 ± 7.0	62.0 ± 14.2	78.1 ± 12.2	70.3 ± 7.2
QUI	88.1 ± 1.6	88.0 ± 2.1	88.7 ± 2.1	88.0 ± 1.8
